# A method for empirically validating FMEA RPN scores in a radiation oncology clinic using physics QC data

**DOI:** 10.1002/acm2.14391

**Published:** 2024-07-10

**Authors:** Leonard H. Kim, Badal R. Juneja, Natalie N. Viscariello

**Affiliations:** ^1^ Department of Radiation Oncology MD Anderson Cancer Center at Cooper Camden New Jersey USA

**Keywords:** FMEA, physics plan review, risk management

## Abstract

In failure modes and effects analysis (FMEA), the components of the risk priority number (RPN) for a failure mode (FM) are often chosen by consensus. We describe an empirical method for estimating the occurrence (O) and detectability (D) components of a RPN. The method requires for a given FM that its associated quality control measure be performed twice as is the case when a FM is checked for in an initial physics check and again during a weekly physics check. If instances of the FM caught by these checks are recorded, O and D can be computed. Incorporation of the remaining RPN component, Severity, is discussed. This method can be used as part of quality management design ahead of an anticipated FMEA or afterwards to validate consensus values.

## 1 INTRODUCTION

In failure modes and effects analysis (FMEA), each failure mode (FM) is assigned numerical values related to the FM's likelihood of occurrence (O), detectability (D), and severity (S). These numbers are multiplied to obtain the FM's risk priority number (RPN), which is used in quality management (QM) to “direct attention to failures that are most in need of QM”.^1^ In published FMEA, values for O, S, and D are often estimated by expert consensus.^1^ ^−^ ^4^ For example, in the “Example Application of TG‐100 Methodology” given in the TG‐100 report, nine members “each supplied an individual estimate of O, S, and D. . . based on their individual experiences. The entire group discussed the evaluations and then pooled them. . .”.^1^ The RPN values given in the TG‐275 Report were obtained similarly.^2^

For a given FM in a given clinic, there are “true” values for O, S, and D, and it is not clear how well they are estimated by either individuals performing that clinic's FMEA or the multi‐institutional consensus values of published reports. We describe here an empirical method for estimating O and D scores in a clinical setting. This can be done ahead of an anticipated FMEA or done afterwards to validate both values independently estimated by a single institution or values adopted from a multi‐institution consensus. We give an example RPN obtained through this method and compare it with the RPN from the TG‐275 report. We also discuss incorporation of S into this method.

## 2 METHODS

### 2.1 Requirements

The method has two requirements. The first requirement is that the analyzed FM has an associated quality control (QC) measure that is performed twice. For the purposes of this paper, we will take physics check to be the QC, and this requirement is thus satisfied if the particular FM is checked for during both initial physics check and the first weekly physics check. This arrangement is specifically described in the TG‐275 report. One of the rationales for weekly physics check given by that report is to detect FMs missed during the initial physics check, and the report's sample first weekly checklist includes “high‐priority failure modes which would benefit from. . . redundant checks”.^2^

The second requirement is recording when a FM is detected by a check and whether it was in the first (e.g., initial physics) or second (e.g., first weekly) check. Though an existing incident learning system (ILS) could be used and mined for this purpose,^5^, ^6^ this may not be optimal compared to a dedicated documentation system where the FM and the check can be entered/selected quickly, easily, and unambiguously. Such a system was used in our clinic and is described elsewhere.^7^

### 2.2 Determination of FMEA parameters, O and D

Given these two requirements, we can define the following quantities that can be used to obtain O and D for a given FM:

$N_{0}$ = # of times FM occurs, regardless of whether it is caught or not.
$N_{1}$ = # of times FM is caught by the first check (e.g., Initial Physics Check).
$N_{2}$ = # of times FM is caught by the second check (e.g., First Weekly Physics Check).
Check Efficacy (CE) = % probability of catching FM in a single check

$N_{1}$ and $N_{2}$ are documented through the second requirement. $N_{0}$ and CE, which are related to O and D, can be expressed in terms of $N_{1}$ and $N_{2}$ as follows:

(1) $$N_{1}=\;N_{0}*CE$$

(2) $$N_{2}=\left(N_{0}-N_{1}\right)\;*CE$$

Re‐arranging (1), substituting for $N_{0}$ in (2), and solving for CE, we obtain:

(3) $$CE\;=\;1-\frac{N_{2}}{N_{1}}$$

Substituting back into (1) and solving for $N_{0}$, we obtain:

(4) $$N_{0}=\frac{{\left(N_{1}\right)}^{2}}{N_{1}-N_{2}}\;$$

Using $N_{0}$ and CE and a few other easily obtained quantities, the following values can be computed and used with Table II from the TG‐100 report to look up values for O and D.^1^

(5) $$\frac{N_{0}}{\#\;of\;plans}$$

This value can be used to look up O in TG‐100 Table II.^1^ The number of plans must be separately obtained.

(6) $${\left(1-CE\right)}^{n}$$

This value can be used to look up D in TG‐100 Table II.^1^ Here, n is the number of QC layers in place to catch the FM. In the example of an initial and first weekly physics check, n is 2.

## 3 RESULTS AND DISCUSSION

### 3.1 Example calculation and interpretation

We emphasize that the computed values given here and later should not be taken as applicable to other clinics without validation from that clinic's own data. Calculations are given for the sole purpose of illustrating our method.

At our clinic, “dose calculation error” (TG‐275 FM# 36) was detected 8 times in initial chart check ($N_{1}$ = 8) and 2 times in the 2nd chart check ($N_{2}$ = 2) over 2358 chart checks. Using Equations (3) and (4), CE for this FM is estimated at 75% and $N_{0}\;$= 11 (i.e., based on the CE, one “dose calculation error” passed through both checks undetected.) Using these values in Equations (5) and (6) and consulting TG‐100 Table II,^1^ we obtain D (with two checks) and O both ≈6. In comparison, TG‐275 consensus values for this FM are D = 4.7 and O = 4.4.^2^ That is, TG‐275 estimated this FM occurs in about 0.15% of plans and goes undetected about 2% of the time, whereas our clinical numbers suggest the FM occurred in 0.5% of our plans and went undetected about 6% of the time.

The next question is whether this calculation would have validated using TG‐275′s values for this FM in our clinic or not. In this case, we consider TG‐275′s value O = 4.4 unlikely to reflect our clinic, because we would then have expected to have recorded 3× fewer instances (i.e., $N_{1}$ around 3 and $N_{2}$ = 0 or 1) of “dose calculation error” in the 2358 plans than we did. If O were really 4.4, our recorded numbers, $N_{1}$ = 8 and $N_{2}$ = 2, would have been very unlikely to occur. In contrast, we do not consider TG‐275′s value D = 4.7 inconsistent with our clinical data, even though it too represents a 3× difference (i.e., 2% vs. 6% undetected). In this case, compared to our actual values, $N_{1}$ = 8 and $N_{2}$ = 2, the expectation values if D = 4.7 would be $N_{1}$ = 8.5 and $N_{2}$ = 1.1. (N.B., this does require O to be 5.7, very close to our observed 5.8), and a larger sample size would be needed for more precision.

### 3.2 Discussion

There are several previous reports relevant to the empirical determination of O and D. Donahue et al. obtained empirical O values for data transfer‐related FMs by analyzing results of an in‐house “automated data comparison software” and used them to improve RPN estimates. However, S and D were still ‘‘determined by either a consensus of the authors or using the values for related items from TG‐275″.^8^ Paradis et al. obtained empirical occurrence rates using their ILS system. One methodical detail in their study is the attempt to account for expected ILS underreporting by assuming event recorders were “80% efficient in recording the events that they caught”.^6^ As noted above, this is why an existing ILS may not be optimal for this purpose.^5^ We used a separate, dedicated recording system designed for ease‐of‐use and with explicit instruction to record everything in an effort to mitigate if not eliminate the underreporting effect.^7^ Siebert et al. describe a process for physics checks consistent with the one described here, including a second review. Perhaps surprisingly, they report, “no errors/deviations were observed” in the second review, implying perfect physics check efficacy and D = 1 for all checked FMs.^9^ Other studies examining physics check efficacy are not so positive. In an ILS analysis, Gopan et al. reported 38% of events passing through physics review were detected, which is equivalent to D = 10.^5^ Gopan et al. later reported on a simulated physics check exercise involving embedded errors. This also suggested D = 10 with a single physics check QC, though some individual FMs were detected at a higher rate, with D as low as 6.^10^

Our technical note expands on this prior work by describing another method to estimate check efficacy and incorporate it into estimates of D and O. This can motivate QM design, especially in determining # of QC layers and efficient checklist creation. For a given FM with associated CE, it follows from Equation (6) that D can be reduced by increasing the number of checks, that is, adding layers of Swiss cheese.^11^ For example, “wrong prescription (energy, bolus)” has a CE of 84% in our clinic. If only one physics check QC is in place, that corresponds to D = 8. However, if checked for three times, for example by being explicitly included in the first weekly check and in an upstream check (a practice advocated by the TG‐100 and TG‐275 reports), D is reduced to below 3, though this should be balanced against the practicality of adding additional formal checks.^12^ In contrast, “wrong target dose” is caught in our clinic with CE effectively 100% (i.e., all recorded instances were caught in initial physics check). If the RPN is considered otherwise acceptable, this FM could be identified as not needing redundant checks and might be justifiably removed from first weekly checklists in the interest of efficiency.

In addition to underreporting, sources of systematic biases include possible error detection outside the designated QCs (e.g., FMs caught prior to physics check as observed in Ref.^6^ and the assumption that the two checks have the same CE, whereas TG‐275 suggests the same nominal check might be performed less rigorously when repeated. Greater accuracy could be obtained by attempting to account for these effects, but we note that O, S, and D already represent broad ranges and may not change as a result. These ranges also provide some cushion against sample size effects, though a large enough sample size is needed at minimum to avoid instances where a FM happens to be recorded more often during the 2nd check than the first, causing Equations (3) and (4) to break down.

There were several reasons physics check was chosen as the exemplar QC for our method. It was expected most clinics already have the multiple layers of physics check needed for implementation, and documentation would be the only added element. In addition, physics check was considered the highest‐yield QC for the method given the relatively large number of FMs it addresses.^13^ Finally, though TG‐275 speculates two physics checks may have different CE, we were more confident they would be similar compared to other candidate QCs. Ford et al. lists a number of QCs that might be candidates for our method, but the uncertainty in the resulting RPN estimates should be considered in each case.^13^ For example, though “physician chart review” and “chart rounds” should both check the FM “Wrong or inaccurate MD contour” (TG‐275 FM# 1), chart rounds might be expected to be less effective in catching the FM, because multiple charts are reviewed in limited time. If data from these two QCs were used, our method would likely underestimate both O and D, because it assumes the two QCs have the same CE. With that caveat, other QCs listed by Ford et al. that might be candidates for our method include chart reviews by non‐physicists (i.e., physician and therapists) and port film and online CT review, as these are typically sequentially performed by multiple people: therapists, physicians, as well as physicists during weekly chart check.

With regard to severity (S), although it is possible to ask that each recorded FM be assigned a value for S in order to obtain an average, this makes the process more onerous and could exacerbate underreporting. In addition, the TG‐100 report observes that (1) severity for detected FMs may not be the same for undetected FMs, and (2) S is not independent of O and D. This interdependence suggests an alternative method: to pre‐define S for a FM (for example, using TG‐275 values) as a threshold for recording. The point of this approach is illustrated in the following example. In our clinic, D and O for “wrong or inaccurate dosimetrist contours” (TG‐275 FM# 7) were both determined to be 10 using our methodology, much higher than the TG‐275 values of 5 and 6 respectively. The discrepancy is explained by differing assumptions about S and how this FM was recorded. In our clinic, any instance of this FM was recorded whether it had clinical impact or not. For example, a single missing interpolated slice of a structure outside the irradiated region would be counted as a “wrong or inaccurate dosimetrist contour.” Our data thus included large numbers of this FM with S in the 1−3 range (“no effect” to “inconvenience”) rather than TG‐275′s value of 6.2 (“limited toxicity or underdose”). Yet, because O, S, and D are multiplied, the RPN ends up being the same despite this discrepancy (i.e., 10 × 10 × 2 is comparable to 5 × 6 × 6.2). On the one hand, the large differences in the individual multipliers end up not mattering because of the interdependence of S with O and D. On the other hand, to facilitate detailed comparisons between FMEA, it would have been preferable to pre‐define Severity in the definition of individual FMs prior to collecting the data to determine O and D.

## 4 CONCLUSION

We present a method to empirically estimate the Occurrence and Detectability parameters used in FMEA as an alternative to the use of consensus values. Options to incorporate Severity into this method are discussed.

## AUTHOR CONTRIBUTIONS

Natalie N. Viscariello initiated the project, collected the data, performed initial analysis, and reviewed and revised the draft. Badal R. Juneja substantially contributed to data collection, analysis, and interpretation, and reviewed and revised the draft. Leonard H. Kim conceived the final version of the project, completed the analysis, and wrote the initial draft.

## CONFLICT OF INTEREST STATEMENT

The authors declare no conflicts of interest.
